# Frequency and Pattern of Heteroplasmy in the Complete Human Mitochondrial Genome

**DOI:** 10.1371/journal.pone.0074636

**Published:** 2013-10-02

**Authors:** Amanda Ramos, Cristina Santos, Ligia Mateiu, Maria del Mar Gonzalez, Luis Alvarez, Luisa Azevedo, António Amorim, Maria Pilar Aluja

**Affiliations:** 1 Unitat d’Antropologia Biològica, Departament de Biologia Animal, Biologia Vegetal i Ecologia, Universitat Autònoma de Barcelona, Cerdanyola del Vallès, Barcelona, Spain; 2 Laboratory of Reproductive Genomics, Department of Human Genetics, KU Leuven, Leuven, Belgium; 3 Institute of Molecular Pathology and Immunology of the University of Porto, Porto, Portugal; 4 Faculty of Sciences of the University of Porto, Porto, Portugal; Ben-Gurion University of the Negev, Israel

## Abstract

Determining the levels of human mitochondrial heteroplasmy is of utmost importance in several fields. In spite of this, there are currently few published works that have focused on this issue. In order to increase the knowledge of mitochondrial DNA (mtDNA) heteroplasmy, the main goal of this work is to investigate the frequency and the mutational spectrum of heteroplasmy in the human mtDNA genome. To address this, a set of nine primer pairs designed to avoid co-amplification of nuclear DNA (nDNA) sequences of mitochondrial origin (NUMTs) was used to amplify the mitochondrial genome in 101 individuals. The analysed individuals represent a collection with a balanced representation of genders and mtDNA haplogroup distribution, similar to that of a Western European population. The results show that the frequency of heteroplasmic individuals exceeds 61%. The frequency of point heteroplasmy is 28.7%, with a widespread distribution across the entire mtDNA. In addition, an excess of transitions in heteroplasmy were detected, suggesting that genetic drift and/or selection may be acting to reduce its frequency at population level. In fact, heteroplasmy at highly stable positions might have a greater impact on the viability of mitochondria, suggesting that purifying selection must be operating to prevent their fixation within individuals. This study analyses the frequency of heteroplasmy in a healthy population, carrying out an evolutionary analysis of the detected changes and providing a new perspective with important consequences in medical, evolutionary and forensic fields.

## Introduction

The distinctive attributes of human mitochondrial DNA (mtDNA), in contrast to the nuclear genome, have turned it into an attractive source of information for population and evolutionary genetics studies. For instance, the high copy number (average of 100–1000 copies per cell) [1] facilitates the study of mtDNA sequences from a wide range of tissue sources, as well as from the partially degraded material found in forensic evidence materials and archaeological remains [2], [3]. Another important attribute of human mtDNA is the maternal inheritance [4], a great advantage in mtDNA studies, as it enables researchers to trace lineages back in time, revealing the evolutionary history of maternal lineages without the complexity of recombination [5]. The mutation and substitution rate of mtDNA, in some orders of magnitude, is higher than that of nuclear genes [6], [7], generating considerable population variability and providing increased resolution of more recent evolutionary events compared to the information provided by nuclear genes.

It is generally assumed that each individual is characterised by a single mtDNA type, but in fact even an isolated cell can harbour a population of distinct mtDNA genomes, a condition known as heteroplasmy. The study of human mitochondrial heteroplasmy goes back to the 1980s, and was first identified in genetic studies of mitochondrial diseases where variable levels of deletions [8] or point mutations [9] were noted in affected patients. However, heteroplasmy is also present in normal individuals. Heteroplasmic variants without apparent functional consequences are observed in samples from individuals without any overt mitochondrial disease [10]–[15]. Thus far, the study of human mtDNA heteroplasmy in healthy individuals has been focused on the analysis of the control region, which only covers 7% of the total mitochondrial genome. Taking into account the functional relevance of the coding region, it is crucial to widen the knowledge of mtDNA heteroplasmy to the whole genome. The few studies that have analysed portions of the coding region of mtDNA in healthy individuals show that heteroplasmy is more frequent in the control region than in the coding region of mtDNA [16]–[18]. Moreover, it has been observed that heteroplasmy levels vary between tissues [10], [11], [15], [19]–[21] and populations [15]. In the analysis of heteroplasmy of the whole mitochondrial genome, several works have been published that focused on the study of diseases or families (e.g. [20], [22]–[24]). However, up until now there are few population studies [18], [25].

Interpreting heteroplasmy as an intermediate stage between the origin and fixation of mutations at the individual or cellular level, implies that it represents an obligatory phase in mtDNA evolution [26]. Thus, it certainly appears to be a useful tool for the study of mutational patterns, the role of selection, and the controversial topic of recombination in mammalian mtDNA [27].

Studies focused on mutations in heteroplasmy at the mtDNA control region have detected important differences between the probability of mutation and the transition/transversion rate [13], [15]. The results of such works suggest that a considerable number of mutations in heteroplasmy are probably eliminated by drift and/or selection. Given that the control region does not code for proteins or RNAs, it is reasonable to assume that less stringent functional constraints act on this portion of the mitochondrial genome. These differences are expected to be much more abundant than in the coding region, and much more relevant from a micro-evolutionary point of view.

Due to sequence similarity between mtDNA and nuclear regions of mitochondrial origin (NUMTs), the interpretation of the mtDNA heteroplasmy could be difficult [28], [29], since co-amplification of mtDNA and nDNA can mimic heteroplasmy. For this reason, a stringent laboratory strategy to address mitochondrial heteroplasmy identification and authentication is mandatory [12], [13], [16], [30], [31].

The main aims of the work presented here are: a) to determine the frequency and pattern of heteroplasmy in the entire mitochondrial genome using an appropriate laboratory strategy for mitochondrial heteroplasmy detection and authentication and, b) to compare the heteroplasmic mutational spectrum with that observed at population level.

## Materials and Methods

### Sample Collection and DNA Extraction

From the 214samples collected and analysed by Alvarez et al. [32], a total of 101 was used in the present study. Alvarez et al. [32] characterised a Northwest Iberian population by sequencing the HVRI segment and informative polymorphisms in the coding region in order to assign each sample to the corresponding mtDNA haplogroup. The samples used in this study represent an equal distribution of genders and haplogroups matching that expected in a Western European population (Table 1). All of the samples were from maternally unrelated Spanish individuals sampled in regional health centres. For all voluntary donors, appropriate informed consent and the birth places of all known maternal ancestors back to the third generation were obtained under strict confidentiality. The present study and the written informed consent were approved by the ethics committee of the Specialized Attention Board at the Healthcare Complex of Zamora and authorised by its Medical Director.

**Table 1:** Haplogroup distribution of 101 analysed individuals.

Haplogroup	Samples	Frequency (%)
H	43	42.58
HV	4	3.96
V	4	3.96
R0	1	0.99
J	13	12.87
K	7	6.93
L	3	2.97
M	1	0.99
N	1	0.99
I	1	0.99
W	2	1.98
T	4	3.96
U	17	16.83
Total	101	

Total DNA from blood was extracted using JETQUICK Blood DNA Spin Kit (Genomed, Löhne, Germany) according to the manufacturer’s specifications.

The information of 37 point heteroplasmies present in 32 individuals and previously published by Li et al. [18] was pooled with our data, for the data analysis.

### MtDNA Analysis

The whole mitochondrial genome was amplified for all samples, using a set of nine primer pairs that prevents NUMTs co-amplification [30], [31]. PCR was performed as described by Ramos et al. [30], [31]. Amplified fragments were purified using the JETQUICK PCR Purification Spin Kit (Genomed, Löhne, Germany). All samples were fully sequenced using 32 internal primers [30], [31]. Sequence reactions were carried out using the sequencing kit Big Dye Terminator v.3 (Applied Biosystems, Foster City, USA) according to the manufacturer’s specifications. Purification of DNA sequencing reactions was carried out with BigDye XTerminator® Purification Kit (Applied Biosystems, Foster City, USA) and sequences were run in an ABI 3130×l sequencer. (Servei de Genòmica, Universitat Autònoma de Barcelona).

### Detection of Heteroplasmy

The sequences obtained were analysed and aligned in relation to the revised Cambridge Reference Sequence (CRS) [33] using SeqScape software (Applied Biosystems, Foster City, USA). A value of 2% in the Mixed Base Identification option was considered in the sequence analysis. This threshold was used as a parameter in the screening software detection, primarily to discard sequences with background and, secondly to automatically identify putative heteroplasmic positions present at low frequencies. Only sequences with satisfactory peak intensity and without background were considered. All sequences were further visually verified and compared with others from the same run in order to establish the presence and level of heteroplasmy.

In order to assess the sensitivity of the approach described to identify mtDNA heteroplasmy, a validation using appropriate controls was performed. Gradient mixtures with minor variants present at 2%, 5% and 10% were generated for positions 71, 9477, 10550 and 16390 of the mtDNA (numbering according [33]). Five independent amplifications were sequenced for each position and percentage. Samples used to perform mixtures were quantified using Quant-iT™ PicoGreen® dsDNA assay. A total of 60 mixtures were analysed using the same procedure of sequencing and heteroplasmy detection previously described for the samples analysed in the present study (Figure S1).

### Authentication of Heteroplasmy

The authentication of mtDNA heteroplasmy was performed following a similar strategy to that used by Santos et al. [12], [13], following three main steps:

1. DNA extraction, PCR amplification, and sequencing of total mitochondrial genome.
2. Sequences revealing heteroplasmy in step 1 were confirmed by a second amplification and sequencing reactions in both senses of the regions where mtDNA heteroplasmy was detected.
3. To authenticate the results and exclude a contamination of the DNA extract for samples presenting heteroplasmy in step 2, an independent DNA extraction, PCR amplification and sequencing in both senses were subsequently performed.

Thus, only point heteroplasmic positions were accepted if they appeared in all validation steps.

Levels of heteroplasmy were determined as described in Santos et al. [12] using the height of peaks in the electropherograms. To calculate the average heteroplasmic levels, the results obtained for at least six sequence reads of each heteroplasmic position were used.

### Data Analysis

#### Haplogroup assignment

Samples were assigned to haplogroups using the online tool HaploGrep [34] following the updated mtDNA phylogeny – mit. Tree build 14 – [35]. Haplotypes presented in this study are available in the EMPOP database (http://www.empop.org) under accession number EMP00555.

#### Statistical analyses

Data published by Li et al. [18] was pooled with our data to increase the statistical robustness. The point heteroplasmy frequencies were estimated by counting, and the Bayesian 0.95 credible region (95% CR) was calculated using the SAMPLING software (V. Macaulay, personal communication).

To calculate the distribution of point heteroplasmy across the mtDNA genome, a proportion test using the OpenStat [36] was performed. The association between haplogroups and point heteroplasmy was evaluated with the Fisher exact test in the Struc program by Genepop 3.3 [37]. The remaining statistical tests mentioned in this work were performed using the program SPSS ver. 15.0.1 software [38].

#### Hits in the phylogeny, population database and Conservation Index (CI)

The number of hits in the phylogeny or the number of occurrences was compiled from the updated mtDNA phylogeny – mit. Tree build 8 – [35] and from Soares et al. [39]. Using the number of occurrences for each mutation reported by Soares et al. [39], the probability of mutation was calculated as the ratio between the observed and the total number of hits. An mtDNA position was considered a hotspot if the mutation probability was ten times higher than the expected mean value. In order to calculate the frequency of each variant for a particular nucleotide position, a database of 3,880 mtDNA complete sequences available from mtDNA phylogeny – mit. Tree build 8 – [35] was created in SPSS format [38] where each mtDNA position represents a variable that allows the calculation of nucleotide frequency.

Nucleotide and amino acid conservation index (CI) were estimated for all heteroplasmic point positions across reference sequences of different Metazoan species. The CI was defined as the percentage of species from the list that had the wild-type nucleotide or amino acid in a given position. A total of 1491 nucleotide sequences and 1628 amino acid sequences were used, respectively, for nucleotides and amino acid CI calculation (for the list of species and accession numbers see Table S1). Sequences were aligned using Clustal W [40] and formatted for further frequency analyses using the SPSS software [38]. Due to the difficulty in obtaining a good alignment for the D-loop, an independent alignment was performed using only primate mitochondrial reference sequences (Table S1).

#### Structure prediction

To understand the structural impact of point mutations found in heteroplasmy, the secondary structure of tRNAs and rRNAs and the prediction of 3D structures of human proteins were performed. The secondary structure prediction was generated using the RNAfold web server [41]. Base-pair probabilities and positional entropy of predicted tRNA structures were used to estimate the implication in the molecule. Human reference tRNA models were compiled [42], but rRNA models are still not available. To circumvent this, additional analysis using the software,mtDNA-GeneSyn version 1.0 [43] was performed to locate rRNA heteroplasmic positions either in the stem or loop region.

To predict the structural impact of non-synonymous substitutions, experimentally determined bovine structures (pdb 1occ and 1bgy for complex IV and bc1, respectively) [44], [45] were used as templates in MODELLER [46]. The software incorporates updated homology modelling methodology [47] for building the human structural models for COI, COII, COIII and CYB. The accuracy of the predicted 3D human models was evaluated in Verify 3D, SOLVX and ANOLEA [48]–[50]. Structural models were visualized in PyMOL [51].

## Results

### Detection and Authentication of Heteroplasmy

Of the 20 sequences of mixtures with the minor variant present at 2%, none was detected by the SeqScape software (Applied Biosystems, Foster City, USA) considering a value of 2% in the Mixed Base Identification option. Similar results were obtained for the 20 sequences presenting a minor variant at 5%. Notwithstanding, the visual inspection of electropherograms demonstrated the presence of base mixture (Figure S1). As for the mixtures with minor variant at 10%, all the 20 sequence mixtures analysed were detected by the software (Figure S1). Thus, for heteroplasmy detection, the sensitivity of capillary electrophoresis, followed by the analysis of sequences with the SeqScape software (Applied Biosystems, Foster City, USA) considering a value of 2% in the Mixed Base Identification option, is 0 for a 2% and 5%, and 1 for a 10% threshold.

According to the criteria for authentication of mtDNA heteroplasmy previously stated in the material and methods section, from all the positions revealing heteroplasmy in the first step only one was removed after a second amplification and sequencing, the remaining heteroplasmic positions were confirmed in all the validation steps.

### Type and Frequency of Heteroplasmy

An exhaustive analysis of frequency and pattern of heteroplasmy was performed for the 101 complete mtDNA genome sequences. A summary of haplogroup frequencies is shown in Table 1 and the complete mutation report of all individuals is available in Table S2. Haplotypes are available in the EMPOP database (http://www.empop.org) under accession number EMP00555.

In this sample collection, 62 individuals (61.39%) presented point and/or length heteroplasmy, and the remaining 38.61% were fully homoplasmic (Table 2).

**Table 2:** Classification of the analysed individuals depending on the heteroplasmic presence.

	Number ofindividuals	Frequency(95% CI)
Homoplasmy	39	38.61 (29.7–48.4)
Heteroplasmy	62	61.39 (51.6–70.3)
1 PH	11	10.89 (6.2–18.5)
1 LH	34	33.66 (25.2–43.4)
>1 PH	2	1.98 (0.6–6.9)
>1 LH	4	3.96 (1.6–9.7)
One or more PH+LH	11	10.89 (6.2–18.5)
Total PH	24	23.76 (16.5–33)
Total LH	49	48.51 (39–58.2)
Total	101	

(PH: point heteroplasmy, LH: length heteroplasmy, CI: confidence interval).

Point heteroplasmy was observed in 23.76% of the individuals. Four individuals presented more than one point heteroplasmy. Heteroplasmy was detected in twenty-seven different positions. With the exception of positions 152 and 16189 that appeared to be heteroplasmic in two individuals, each position was heteroplasmic in a single individual. The frequency of point heteroplasmy did not show significant differences between genders (Chi-squared test: χ^2^ = 0.523, df = 1 *P* = 0.47), nor was it associated with any particular mtDNA haplogroup (Fisher exact test: *P* = 0.442).

As regards length heteroplasmy, 48.51% of the individuals were heteroplasmic. In this case, 34 individuals had only one length heteroplasmy, and the remaining carried more than one length and/or one point heteroplasmy (Table 2). The regions of the mtDNA genome showing length heteroplasmy are presented in detail in Table 3. The highest frequencies of length heteroplasmy were observed in the common poly-C or poly-AC tracts of the control region, with the poly-C-stretch of HVR II (between positions 303–315 of the mtDNA) having the most, with70.9%. Otherwise, three individuals had length heteroplasmy in different regions of mtDNA: between positions 8272–8278, 956–965, and in position 8289 (Table 3).

**Table 3:** Distribution of length heteroplasmy along the mtDNA genome.

	Number of heteroplasmies	Frequency (95% CI)
HVRI poly-C (16184–16193)	5	9.1 (4–19.6)
HVRII poly-C (303–315)	39	70.9 (57.8–81.2)
HVRIII		
poly-AC (514–525)	3	5.4 (2–14.9)
poly-C (568–573)	5	9.1 (4–19.6)
Coding region		
956–965	1	1.8 (0.4–9.6)
8272–8278	1	1.8 (0.4–9.6)
8289	1	1.8 (0.4–9.6)
Total	55	

### Pattern and Distribution of Point Heteroplasmy Along the mtDNA Genome

A total of 29 point heteroplasmic positions were confirmed in our study. To obtain a more complete pattern of point heteroplasmy, data published by Li et al. [18] was pooled together with our data (for a detailed list of positions see Table S3), since the distribution of heteroplasmy across the mtDNA genome is similar between the two data sets, and the sensitivity of two sequencing techniques is also similar.

In order to determine if point heteroplasmy followed a differential distribution along the mitochondrial genome, tests for difference between two independent proportions were performed, and statistical significant differences (after Bonferroni correction) were only observed between control and coding region, with the control region being the most frequent location of mtDNA heteroplasmy (Proportion test: z = 7.817, *P*<0.0001).

The point heteroplasmy distribution is shown in Table 4. Overall, a frequency of 31.82% was observed in the control region, 7.58% in tRNAs, 4.5% in rRNAs, and 56% in protein coding genes positions. Among the 37 point heteroplasmies in coding gene positions, 16 mutational events imply amino acid replacements (43.24%) resulting in a non-synonymous:synonymous ratio of 1∶1.3 (Table 5).

**Table 4:** Distribution of point heteroplasmy along the mtDNA genome regions in the present study and reported by Li et[18].

mtDNA region	Present study		Li et al. [18]		Pooled Data	
	Number ofheteroplasmies	Frequency(95% CI)	Number ofheteroplasmies	Frequency(95% CI)	Number ofheteroplasmies	Frequency(95% CI)
D-loop	8	27.6 (14.2–44.6)	13	35.1 (21.8–51.4)	21	31.8 (21.5–43.3)
HVRI	3	10.3 (3.6–25.8)	5	13.5 (6–28.1)	8	12.1 (6.2–21.9)
HVRII	5	17.2 (7.5–33.7)	8	21.6 (11.4–37.3)	13	19.7 (11.7–30.5)
Coding region	21	72.4 (54.1–85.3)	24	64.9 (48.6–78.2)	45	68.2 (56.2–78.2)
tRNA	4	13.8 (5.5–29.8)	1	2.7 (0.6–13.8)	5	7.6 (3.3–16.3)
rRNA	1	3.4 (0.8–17.2)	2	5.4 (1.7–17.7)	3	4.5 (1.7–12.5)
Complex I	7	24.4 (3.4–13.6)	14	37.8 (24–54)	21	31.8 (21.5–43.3)
Complex III	2	6.9 (2–21.4)	3	8.1 (2.9–21.4)	5	7.6 (3.3–16.3)
Complex IV	4	13.8 (55.4–85.8)	2	5.4 (1.7–17.7)	6	9.1 (4.2–18.2)
Complex V	3	10.3 (3.6–25.8)	2	5.4 (1.7–17.7)	5	7.6 (3.3–16.3)
Total	29		37		66	

**Table 5:** Comparison of results obtained in present study with heteroplasmy detected by Santos et[16] and with the whole mtDNA genome mutational spectrum at population level [43].

Type of mutation	Heteroplasmy inpresent study^a^	Heteroplasmyin D-loop^b^	Mutational spectrumat population level^c^
Transition:Transversion			
Whole mtDNA genome	15.5∶1	–	7.5∶1
Control Region	21∶0	57.5∶1	20.8∶1
Purine:Pyrimidine			
Whole mtDNA genome	1.14∶1	–	1.28∶1
Control Region	1∶3.2	1∶2.93	1∶1.7
Non-synonymous:Synonymous	1∶1.3	–	1∶1.97

a Pooled data of present work and by Li et al. [18].

b [16].

c Computed using data of mtDNA mutation fixed at the individual level and polymorphic at population level, by Pereira et al. [43].

Concerning the type of mutation, the 33 point mutations detected in heteroplasmy were purine transitions and 29 pyrimidine transitions, and only 4 transversions were detected. The transition:transversion and purine:pyrimidine ratios are also shown in Table 5.

### Stability of Point Heteroplasmic Positions

Additional analyses were performed on the 66 point heteroplasmies found in 60 different positions of mtDNA genome to predict the impact of mutation. For this, the number of hits in the mtDNA phylogeny, the probability of mutation, the frequency in the population database, and the conservation index (CI), both at nucleotide and at amino acid level, were calculated, and results are presented in Table S3.

**Figure 1:**
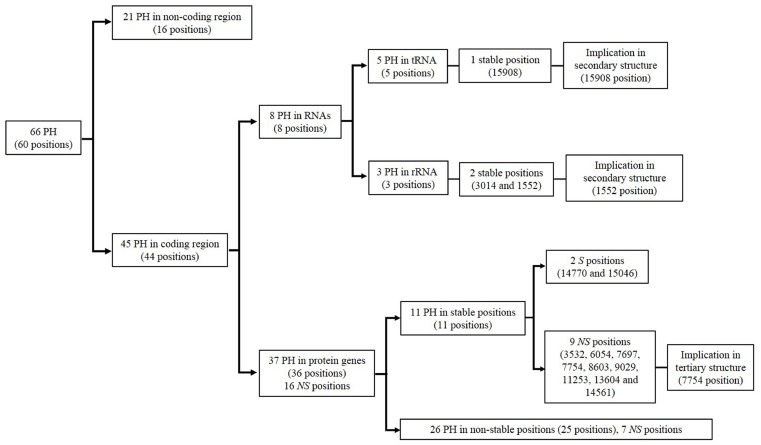
Schematic information of point heteroplasmies analysed in the present study and reported by Li et al. [**18**] **.** Information about distribution along the mtDNA genome, kind of mutation (synonymous/non-synonymous), stability^a^ of positions and implication in secondary and tertiary structure is reported. (PH: point heteroplasmy, *S*: synonymous, *NS*: non-synonymous). ^a^ Defined by: distribution in database population, number of hits in the phylogeny and nucleotide and amino acid conservation index.

A total of 21 point heteroplasmies were located in 16 different positions of the non-coding region (Fig. 1 and Table S3). These heteroplasmic positions have typical characteristics of non-stable position: high number of hits in the phylogeny, high frequency of the minor variant in the population database, and low conservation index.

As regards the coding region, 8 point heteroplasmies (in 8 positions) were located in the stem regions of mitochondrial rRNA and tRNA (Table 4). Of these, 2 heteroplasmic positions (1552 and 3014) were highly stable and conserved. These positions do not appear in the mtDNA phylogeny or in the population database, and have a nucleotide CI over 70% (Fig. 1 and Table S3). Thirty seven point heteroplasmies were located in 36 different positions of protein coding genes (Fig. 1 and Table S3). There were a total of 11 point heteroplasmies in 11 stable positions (3532, 6054, 7697, 7754, 8603, 9029, 11253, 13604, 14561, 14770, and 15046). All these 11 positions had a maximum of two hits in the phylogeny, a minimum of 99.6% of representation in database population as regards the major variant, and a minimum nucleotide CI of 77.11%. Moreover, nine mutational events in stable positions represented non-synonymous mutations (3532, 8603, 6054, 7697, 7754, 9029, 11253, 13604, and 14561). From the total of 11 point heteroplasmies, six of them can be considered as highly stable positions (6054, 7697, 7754, 9029, 13604, and 15046). Besides the characteristics mentioned above (low number of hits, low representation in database population, and high CI at nucleotide level), these positions present CI ≥92.9% at amino acid level (Table S3).

A classification based on the stability of point heteroplasmic positions is shown in Table 6. A similar classification was made for the hits in the phylogeny in order to determine the distribution of fixed mutations at population level [39]. The proportion of point heteroplasmic positions in hotspots was similar to that observed at population level. Interestingly, there was an excess of point heteroplasmies located in positions with no hits in the phylogeny.

**Table 6:** Distribution of point heteroplasmies and the hits in the phylogeny considering the stability of position.

mtDNA positions	Point heteroplasmy		Fixed mutations at population level^c^	
	*N*	Frequency (95% IC)	*N*	Frequency (95% IC)
0 Hits^a^	12	18.18 (10.8–29.2)	0	0 (0.00–0.00)
Hotspot^b^	29	43.28 (32.1–55.2)	4089	38.29 (37.8–38.7)
No hotspot ≥ mean	8	11.94 (6.5–21.9)	2872	26.89 (26.1–27.7)
No hotspot < mean	17	25.37 (16.5–37)	3719	34.82 (33.9–35.7)

(Mean value of the probability of mutation^a^ = 6.034×10^−5^). *N*: total number of mutations.

a Probability of mutation: number of hits/total number of hits.

b Hotspot: probability of mutation ten times higher than the mean value.

c Number of mutations by position based on the mitochondrial phylogeny from Soares et al. [39].

To identify the impact of mutation on the stability of secondary and tertiary conformation of tRNA, rRNA and proteins encoded by mtDNA, a prediction of different structures with the wild type and mutant variant was performed, and those that are likely to imply changes in secondary or tertiary structures are then presented (the set of figures for secondary structure prediction are given in Figure S2).

**Figure 2:**
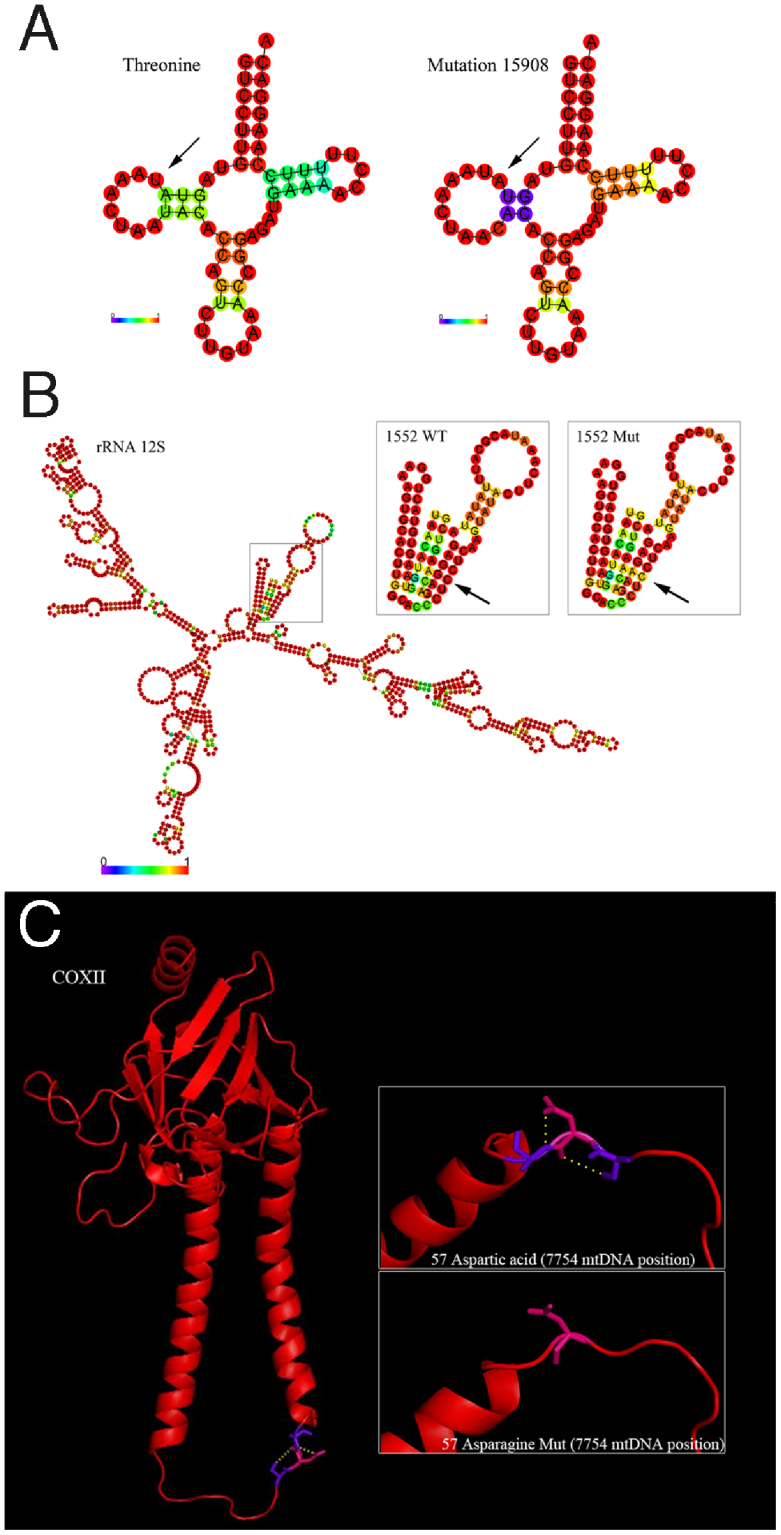
Secondary and tertiary structures prediction. The secondary structure below is coloured by base-pairing probabilities, for unpaired regions the colour denotes the probability of being unpaired. Structure drawing encoding positional entropy is reported in Figure S2. (WT: wild-type, Mut: mutated). (**a**) Secondary structure prediction of threonine tRNA and implication of point heteroplasmic position 15908. (**b**) Secondary structure prediction of 12S RNA and implication of point heteroplasmic position 3014. Detailed view of wild-type and mutated position is showed. (**c**) Three-dimensional model of human COXII complex. Detailed H-bond differences between the wild-type Asp57 and the mutated Asn57 due to a point heteroplasmic position 7754 are presented.

In the case of tRNAs, it seems that point heteroplasmic position 15908 located in threonine tRNA implies a reduction in the number of residue-residue bonds that involve one of the stems of this tRNA (Fig. 2a). Moreover, this position presents two hits in the phylogeny, a representation of a minor variant of 0.1% in the population database, representing a relatively stable position (Table S3). Two positions in the rRNAs located in 12S and 16S are in stem regions (1552 and 3014), and the remaining one in the loop region (2887). The heteroplasmy located in 1552 position implies a reduction in the stability of bonds of this stem region. Moreover, entropy information of bonds in this position is reduced (Fig. 2b).

As for the implications in the tertiary structures of proteins, experimentally determined structures were only available for complex IV and cytochrome bc1 complex. Four non-synonymous heteroplasmic positions (6052, 7697, 7754, and 15314) were located in these regions (the set of figures for tertiary structure prediction are given in Figure S2 and Fig. 2c). Structural predictions indicated that the amino acid substitution (Asp57Asn) in COXII region at heteroplasmic position 7754 might be involved in the interactions between amino acids in the tertiary structure. The mutated amino acid is located in the centre of an α-helix, and the substitution of an acidic by a polar amino acid implies the loss of interactions with the neighbouring amino acids, Ser56 and Ala58 (Fig. 2c). Moreover, as reported above, the heteroplasmic position 7754 is one of the most conserved and stable positions (Table S3).

## Discussion

### Frequency and Type of Heteroplasmy

The frequency of heteroplasmy at population level has been estimated for the hypervariable regions of mtDNA [13], [15], [52] and compared with the previously published study on mtDNA heteroplasmy using complete mitochondrial genomes [18].

In the present study, 61.39% of individuals were heteroplasmic: 48.51% of individuals presented length heteroplasmy, and 23.76% of individuals were carriers of point heteroplasmy. The frequency of length heteroplasmy obtained in the control region [4.95% for HVRI (95% CI, 2.2–11.1%), and 38.61% for HVR II (95% CI, 29.7–48.4%)] was significantly lower than that reported by Santos et al. [13] [17.145% for HVRI (95% CI, 12.3–22.93%), and 64.76% for HVR II (95% CI, 57.89–71.21%)]. These differences could not be due to methodological approaches, since both studies were performed using similar methodology and analytical conditions. It is possible that the differences could be related to the population of origin, since other authors reported frequency differences in point heteroplasmy in different populations [15].

Concerning the frequency of individuals with point heteroplasmy in the control region, our values [7.9% (95% CI, 4.1–14.9%)] are slightly higher than that reported by Santos el at. [13] [3.81% (95% CI, 1.66–7.37%)], however the differences were not significant. No differences were observed on comparing our results of point heteroplasmy frequency [23.76% (95% CI, 16.5–33%)] for the total mtDNA genome with those of Li et al. [18] [24.42% (95% CI, 17.9–32.5%)]. Our results demonstrate the high frequency of heteroplasmy at the population level.

Different technologies have been used for heteroplasmy detection. The majority of works published so far have used the automated Sanger sequencing, the same method used in the present study. On the other hand, next-generation sequencing has been used by Li et al. [18]. Although the sequencing method is different, both studies report a similar level of sensitivity (10% threshold). Moreover, similar point heteroplasmy frequencies and distribution of point heteroplasmy across the mtDNA genome have been detected in both studies. In the present study, special attention was taken to avoid co-amplification between mtDNA and nDNA, as well as an efficient protocol to validate point heteroplasmy. Despite this, and in agreement with others [13], our study demonstrates that low heteroplasmy levels can be detected with confidence using the automated sequencing system, if a good sequencing strategy and an accurate procedure of heteroplasmy detection and validation are used. Moreover, comparing our results with that reported by Li et al. [18], it can be inferred that the methodology used in the present study could be as precise as massive sequencing.

No significant differences were found in the distribution of point heteroplasmy between genders or haplogroup, a result similar to that obtained by Camargo et al. [52]. These results suggest that evolutionary forces that control the levels of heteroplasmy act independently of these two aspects.

### Pattern and Stability of Point Heteroplasmic Positions Along the mtDNA Genome

Transition variants were involved in 94% of point heteroplasmic positions, yielding a transition:transversion ratio of 15.5∶1 (Table 5). The high proportion of transitions is in agreement with the mutational spectrum at population level reported by Pereira et al. [43] (ratio 7.5∶1). However, in the present study there are twice as much transitions in heteroplasmy than at the population level. More than half of them involved purines (53.2%), representing a purine:pyrimidine ratio of 1.14∶1, in agreement with the 1.28∶1 ratio at population level [43]. In agreement with previous reports that consider mutations in heteroplasmy and at population level [13], [15], [43], opposing proportions were detected in the control region, where a high proportion of mutations involving pyrimidines (76.2%) were detected (Table 5). This discrepant result between mitochondrial regions cannot be due to differential base composition, since there is a similar purine:pyrimidine ratio (1∶1.31) in both regions, and thus the causes of such difference deserve further investigations.

Mutations are the root cause of heteroplasmy, and also provide the basis for sequence evolution, so it is reasonable to expect a correlation between sites that experience high rates of heteroplasmy and those that evolve quickly within populations [15]. However, it seems that a different pattern is established for point heteroplasmy, since there is an excess of point heteroplasmies located in positions that present 0 hits in the phylogeny.

A total of 16 different point heteroplasmies were detected in non-synonymous positions, representing a non-synonymous:synonymous ratio of 1∶1.3. These results are similar to the values reported by others at the population level (ratio 1∶1.97) [43]. Pereira et al. [43] found high levels of non-synonymous polymorphisms in ATP6 and ATP8 regions of mtDNA, and low levels in COI, ND4 and ND5 regions. In the present work, the distribution between regions is different, probably due to the low number of non-synonymous positions detected. The same authors showed that the group of neutral amino acids (Valine, Isoleucine, Alanine, Methionine, and Threonine) present the highest proportion of all possible changes observed in the human population. Our data shows that out of 16 non-synonymous heteroplasmic positions, seven corresponded to a replacement to an amino acid of the same group, and all imply an amino acid change with the same polarity/acidity (neutral to neutral), suggesting that the new residue could maintain the interactions with the other residues in order to preserve the tertiary structure of the protein.

One of the most conserved positions in the tRNAs where heteroplasmy was found was 15908. This change implies a reduction in the stability of the molecule due to a length reduction of one of the stems of the threonine tRNA. It has been reported that some polymorphisms in the human population are located in positions identified as 100%, and >90% conserved in mammalian tRNAs [53]. Moreover, Pereira et al. [43] and Kivisild et al. [53], found threonine tRNA as the one with the highest number of polymorphisms. Along with the point heteroplasmic position 15908, there are four more heteroplasmies located in tRNAs, all of them found in stem regions. These results are in contrast with Pereira et al. [43], who found an equal stem:loop ratio, making sense with expectations based on the key role of stem regions in the maintenance of the secondary structure.

Eleven point heteroplasmies detected in protein coding genes were located in stable positions, presenting a representation in mtDNA phylogeny near zero, low or null levels of minor variant in the population database, and high levels of CI. Moreover, from those heteroplasmic positions located in complexes for which the mitochondrial crystal structures is known, the 7754 position, which involves an amino acid substitution Asp70Asn, has a direct implication in the tertiary structure of COXII, implying the loss of interactions with nearby amino acids.

In the present study, 12 point heteroplasmic positions did not show variability at population level (presenting zero hits in the mitochondrial phylogeny) and the implication in mitochondrial pathologies is unknown. However, two stable positions (7697 and 11253, both presenting 2 hits in the mitochondrial phylogeny) have been related to mitochondrial pathologies.

The point heteroplasmy located in 7697 position, and the implication of the amino acid change (Val<Iso) has been reported as a genetic factor of susceptibility to Hypertrophic cardiomyopathy (HCM) in Chinese Han ethnic population [54]. As regards the point heteroplasmy located in 11253 position (Iso<Thr), this has a direct implication on Leber’s hereditary optic neuropathy (LHON) [55], [56]. Even though the frequency of the mutant variant of this individual is reasonably high (76%), in most of LHON patients, the pathogenic mtDNA mutation is homoplasmic [57], [58].

As previously mentioned, most of mtDNA heteroplasmies comprise transitions. It seems that evolutionary forces may be acting to lower them at population level. Most probably, these mutations will be finally removed by genetic drift or by selection. In fact, all of the stable point heteroplasmic positions could have a greater impact in the viability of mitochondria survival, suggesting that purifying selection could be operating on some heteroplasmies to prevent their fixation within individuals. Although purifying selection have been proposed by others [13], [26], [59], [60], no previous evidence involving heteroplasmy has been found, but in accordance with Li et al. [18], currently there are not enough heteroplasmic studies to evaluate the role of purifying selection.

This study analyses the frequency of heteroplasmy in healthy population, carrying out an evolutionary evaluation of the detected changes. We believe that this study is starting to change this scenario by providing a new perspective that is important for medical, evolutionary, and forensic purposes.

## Supporting Information

Figure S1 **Electropherogram of the gradient mixtures with minor variants present at 2%, 5% and 10% proportions in position 10550 (A/G).** Results obtained by SeqScape software considering a value of 2% in the mixed base identification option. Results from sample mixtures at 2% and 5% do not show differences from the reference sequence, while sample mixture at 10% reported a mixed base of A/G. Nomenclature used according to IUPAC.
(PDF)

Figure S2 **Implication of mtDNA point heteroplasmies in the secondary structure of tRNAs, rRNAs and tertiary structure of COXI, COXII and CytB.**
(PDF)

Table S1 **List of accession number and species used for the conservation index estimations at nucleotide level.**
(XLSX)

Table S2 **Mutation report of complete mitochondrial DNA of 101 individuals.** Only base change in transversions is showed. Point and length heteroplasmy are showed in bold. Sex and haplogroup is also reported.
(XLSX)

Table S3 **Complete results of each heteroplasmic position analyzed (map locus, position, sample name, heteroplasmy type, heteroplasmy origin, mean proportion of height peaks, distribution in population database, number of hits in mtDNA phylogeny (PhyloTree.org) and by Soares et al.** [**39**] **, probability of mutation and nucleotide and amino acid Conservation Index).**
(XLSX)
