# Supplementary material for: Frequency and Pattern of Heteroplasmy in the Complete Human Mitochondrial Genome
Source: PLoS One. 2013 Oct 2;8(10):e74636. doi: 10.1371/journal.pone.0074636 (PMC3788774; doi:10.1371/journal.pone.0074636)

**Supplementary Figure S1.** Electropherogram of the gradient mixtures with minor variants present at 2%, 5% and 10% proportions in position 10550 (A/G). Results obtained by SeqScape software considering a value of 2% in the mixed base identification option. Results from sample mixtures at 2% and 5% do not show differences from the reference sequence, while sample mixture at 10% reported a mixed base of A/G. Nomenclature used according to IUPAC.

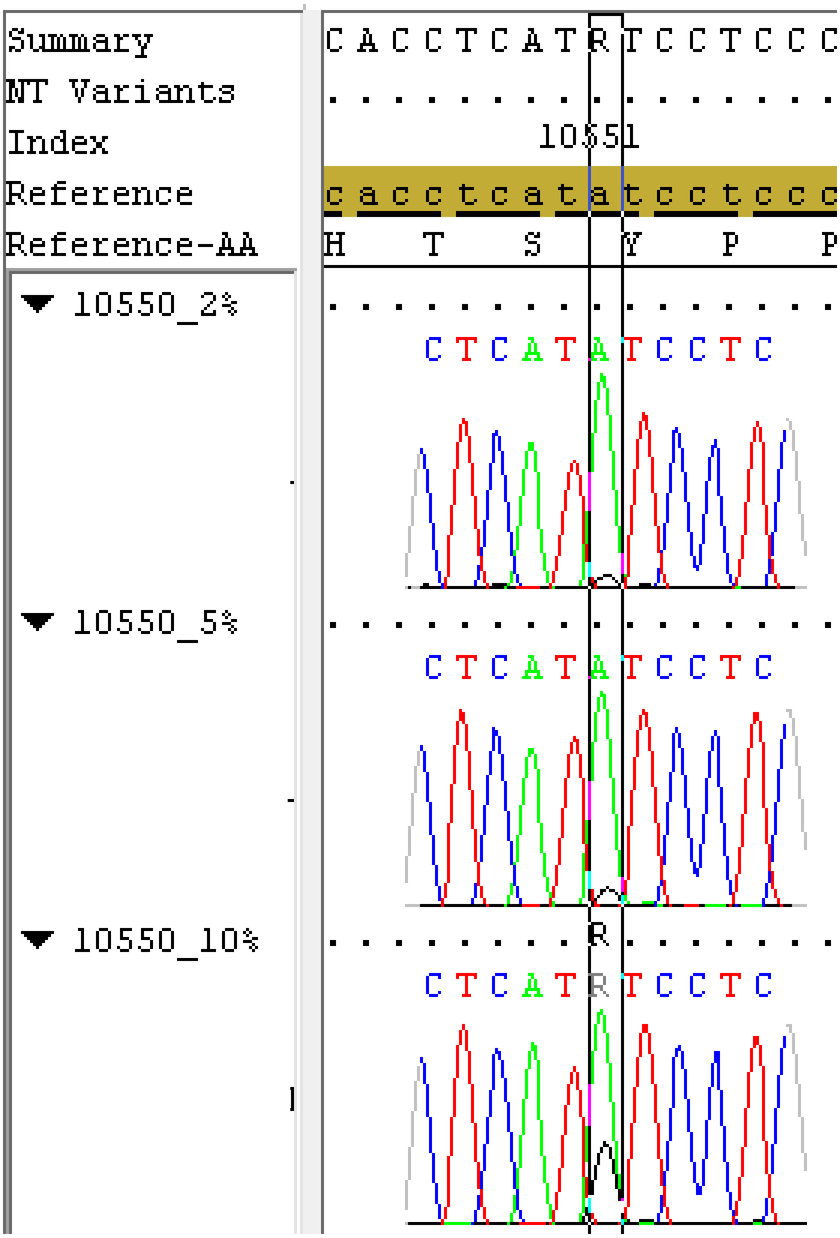

Supplement: Figure S1 — Electropherogram of the gradient mixtures with minor variants present at 2%, 5% and 10% proportions in position 10550 (A/G). Results obtained by SeqScape software considering a value of 2% in the mixed base identification option. Results from sample mixtures at 2% and 5% do not show differences from the reference sequence, while sample mixture at 10% reported a mixed base of A/G. Nomenclature used according to IUPAC. (PDF) [file pone.0074636.s001.pdf]
