# Supplementary material for: Frequency and Pattern of Heteroplasmy in the Complete Human Mitochondrial Genome
Source: PLoS One. 2013 Oct 2;8(10):e74636. doi: 10.1371/journal.pone.0074636 (PMC3788774; doi:10.1371/journal.pone.0074636)
Supplement: Figure S2 — Implication of mtDNA point heteroplasmies in the secondary structure of tRNAs, rRNAs and tertiary structure of COXI, COXII and CytB. (PDF) [file pone.0074636.s002.pdf]

Supplementary Figure S2. Implication of mtDNA point heteroplasmies in the secondary structure of tRNAs, rRNAs and tertiary structure of COXI, COXII and CytB

tRNA Threonine entropy

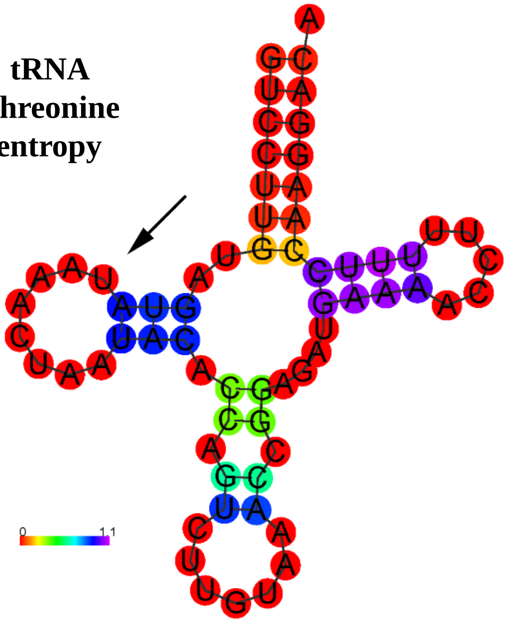

Mutation 15908 entropy

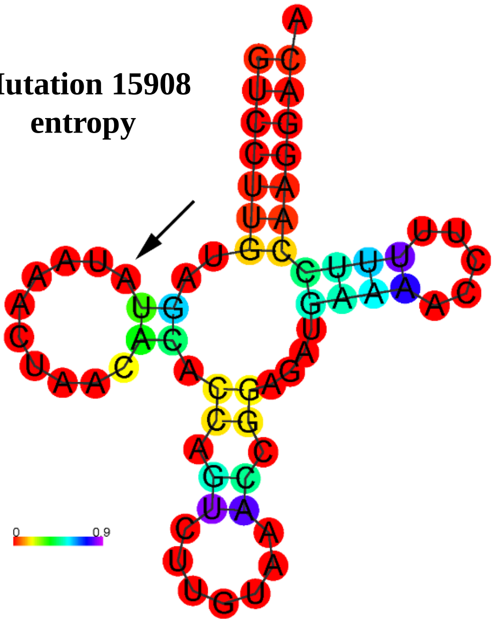

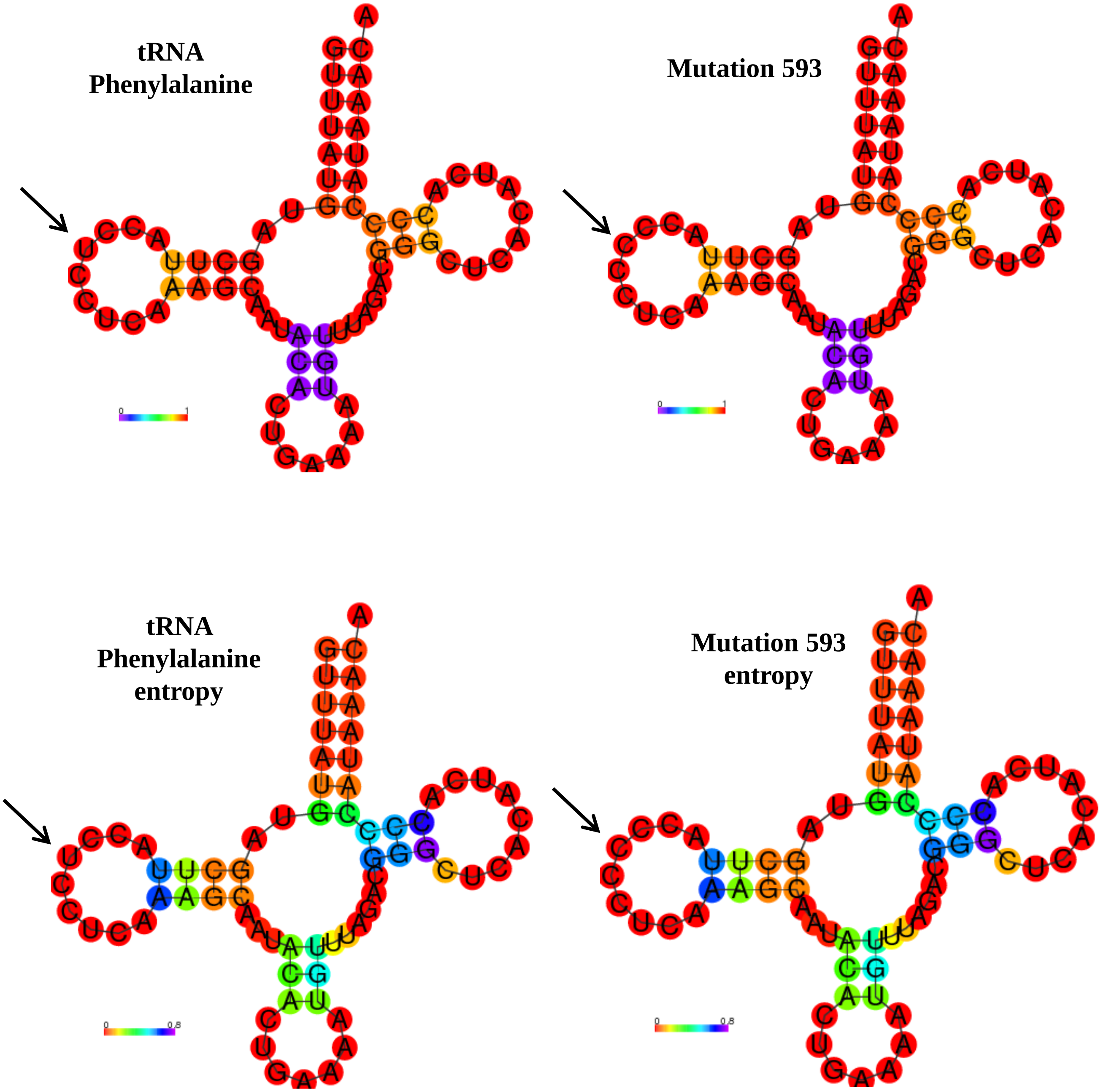
tRNA
Phenylalanine
Mutation 593
tRNA
Phenylalanine
entropy
Mutation 593
entropy

tRNA Lysine

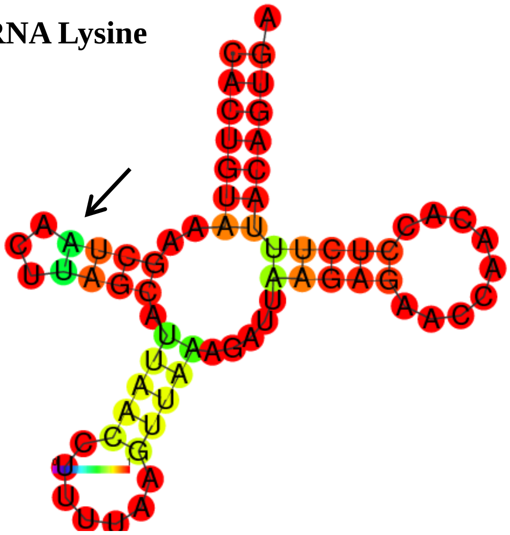

Mutation 8307

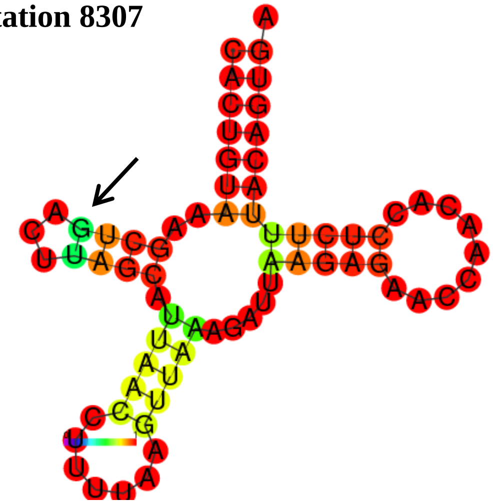

tRNA Lysine entropy

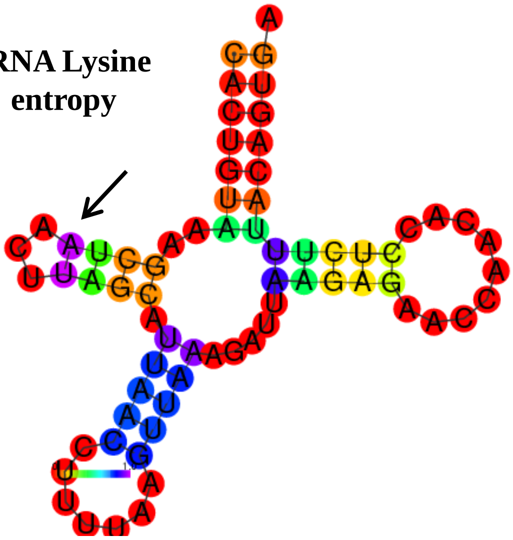

Mutation 8307 entropy

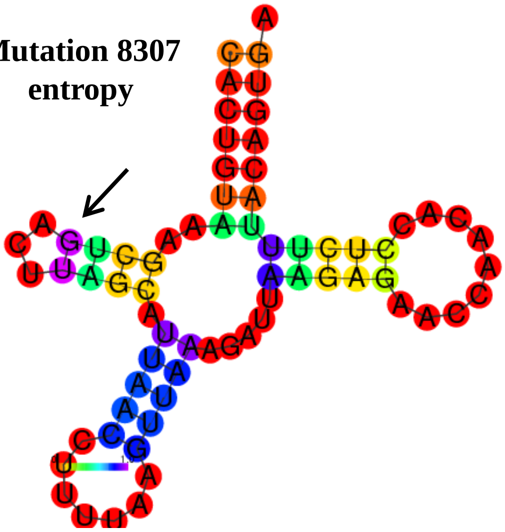

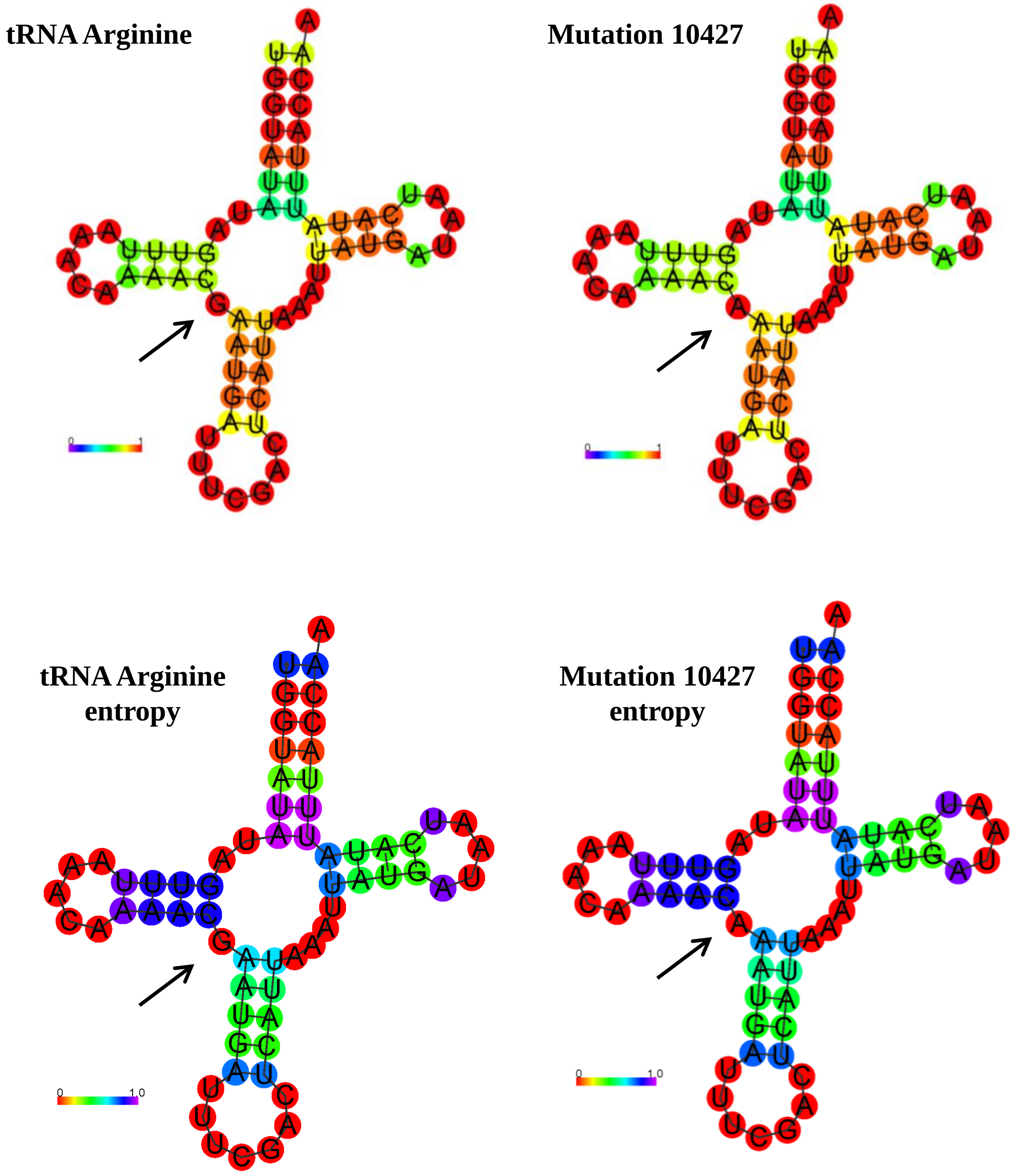
tRNA Arginine
Mutation 10427
0
1
tRNA Arginine entropy
Mutation 10427 entropy
0
1.0

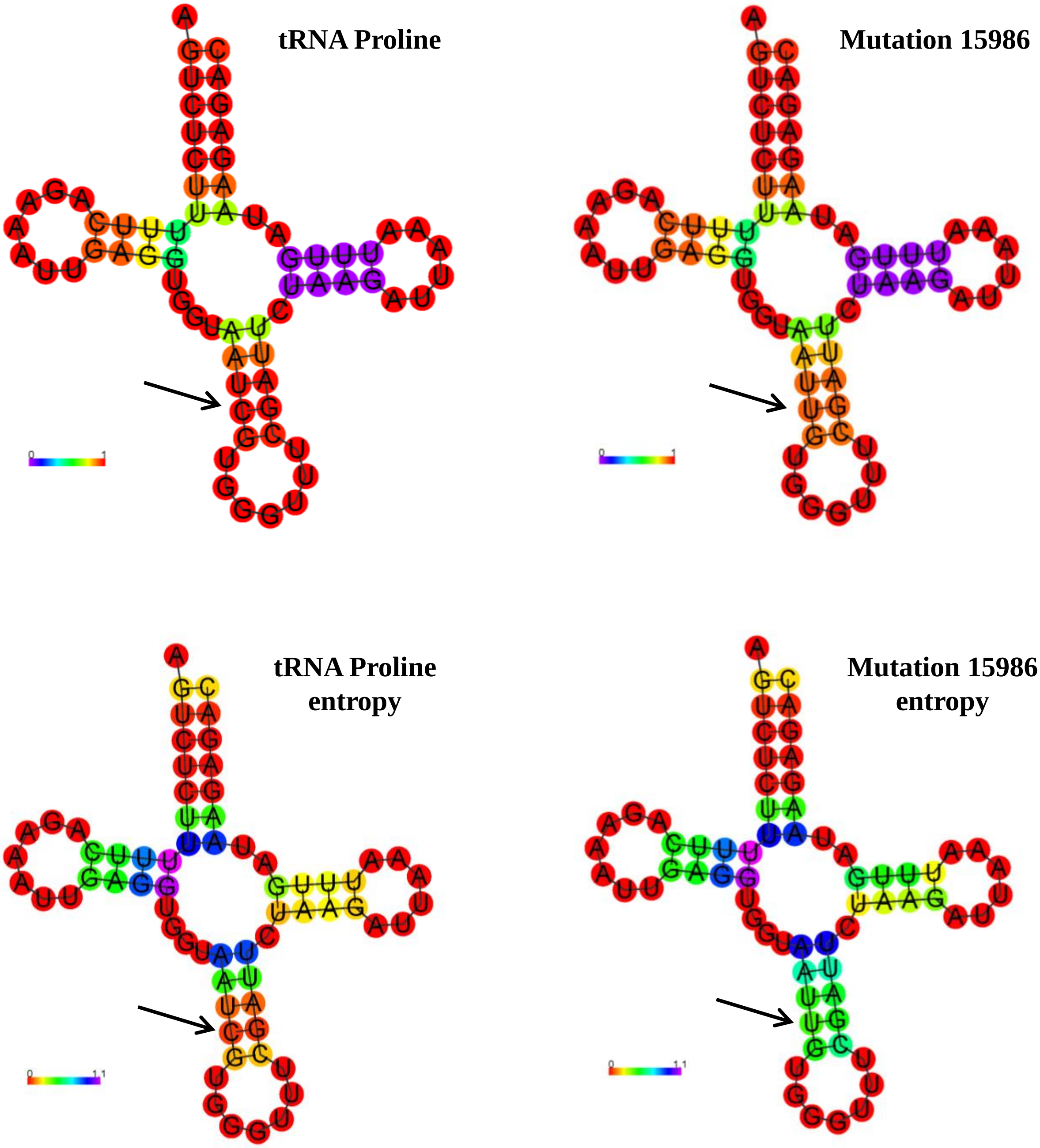
tRNA Proline
Mutation 15986
tRNA Proline entropy
Mutation 15986 entropy
0
1
0
1.1

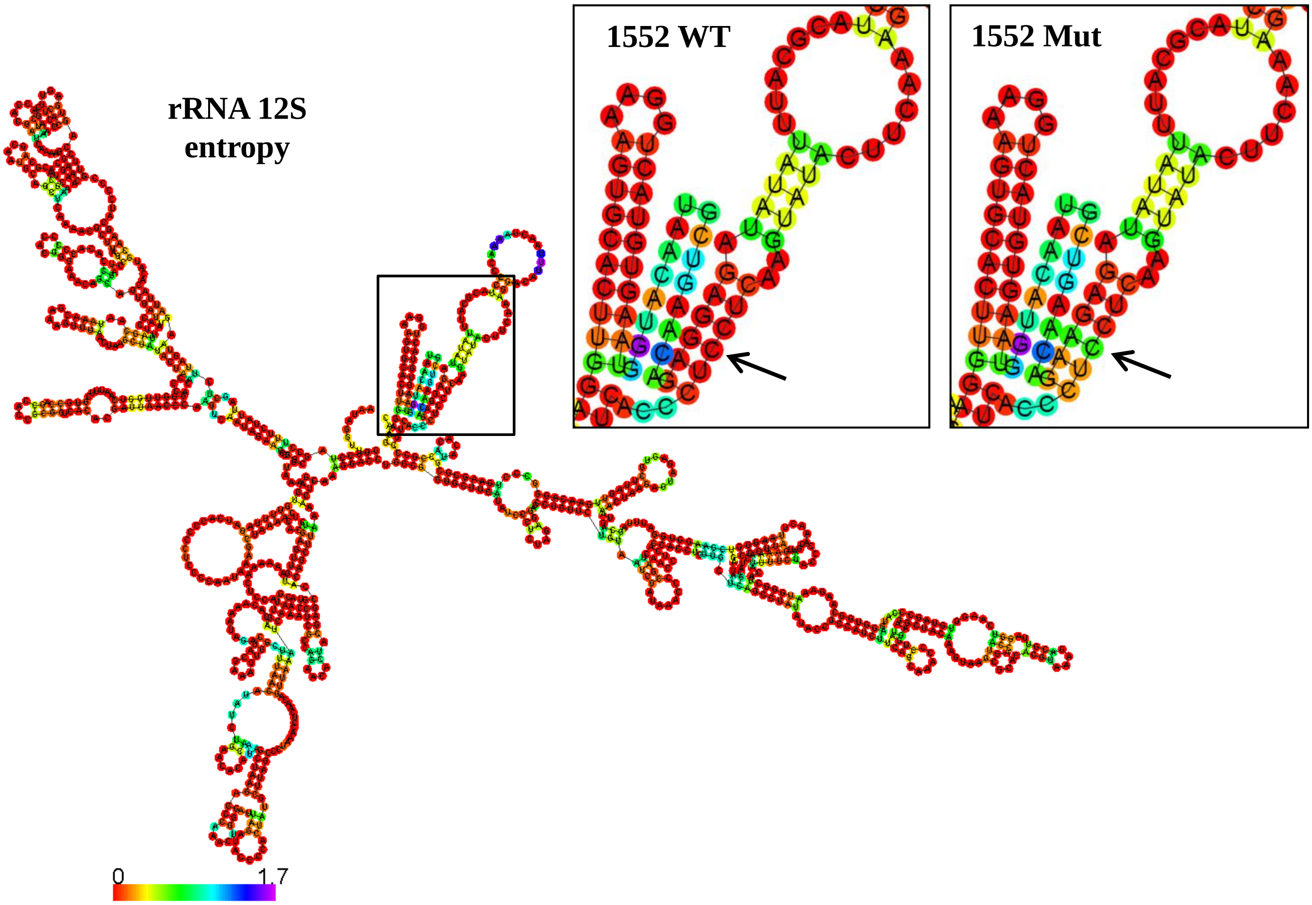
rRNA 12S
entropy
0
1.7
1552 WT
1552 Mut

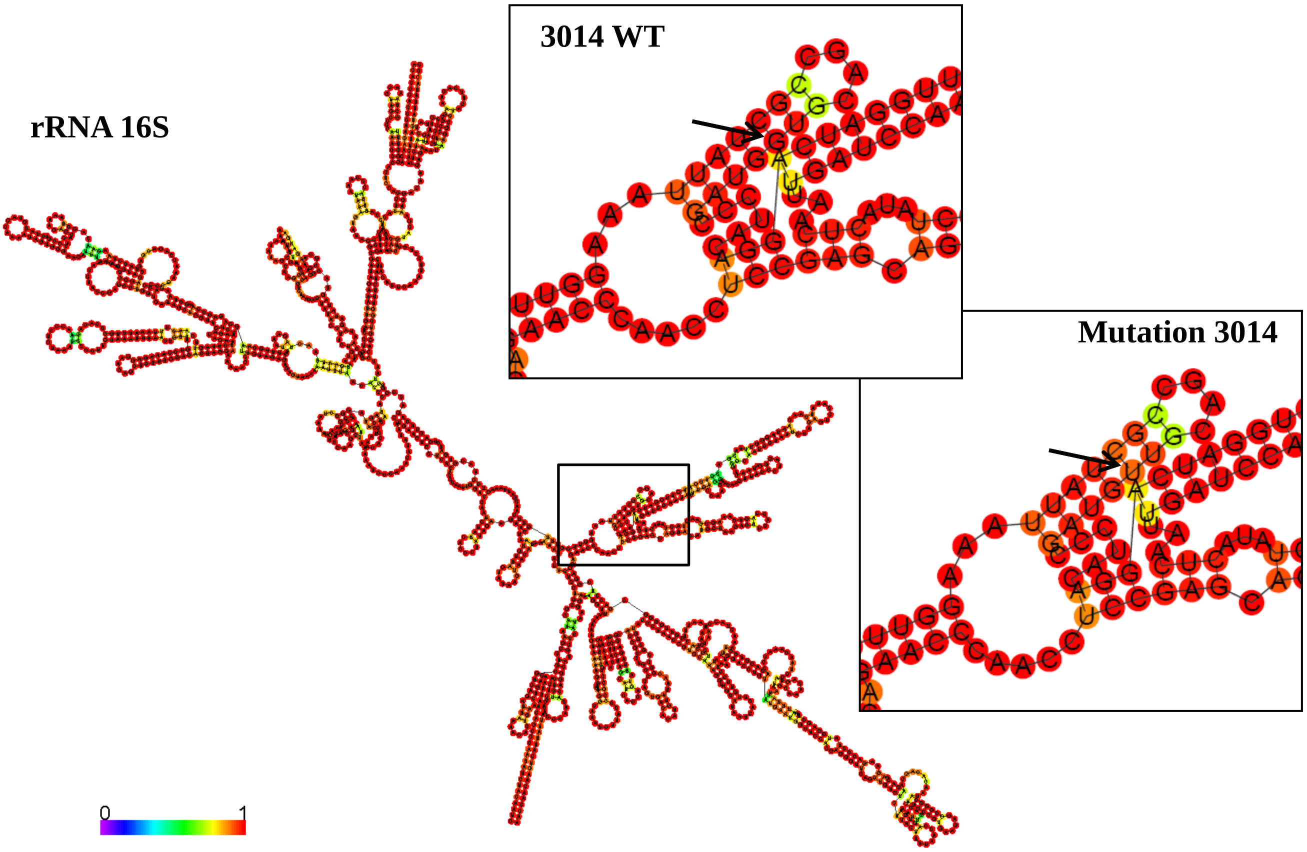
rRNA 16S
3014 WT
Mutation 3014
0
1

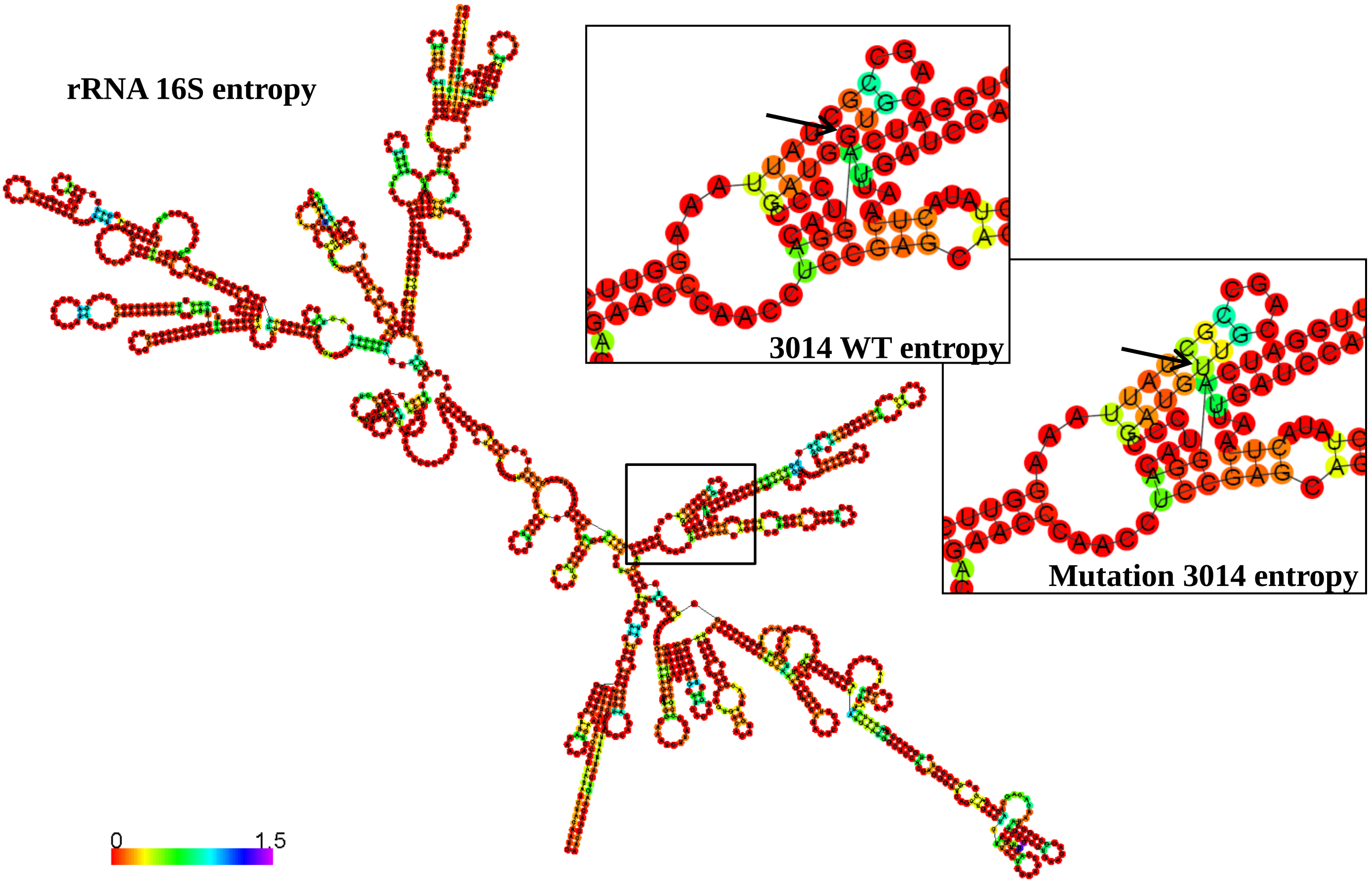
rRNA 16S entropy
3014 WT entropy
Mutation 3014 entropy
0
1.5

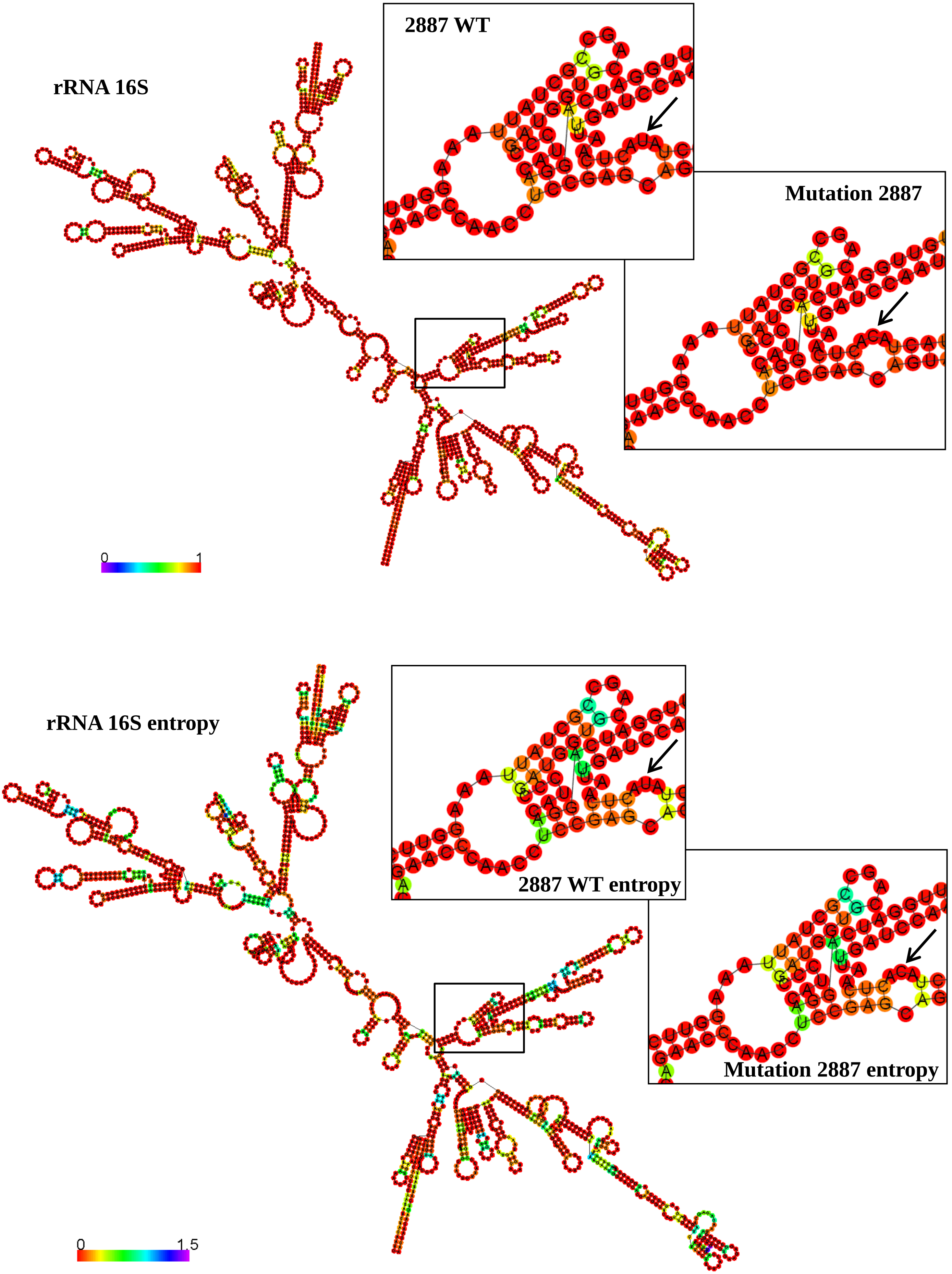

rRNA 16S
2887 WT
Mutation 2887
0
1
rRNA 16S entropy
2887 WT entropy
Mutation 2887 entropy
0
1.5

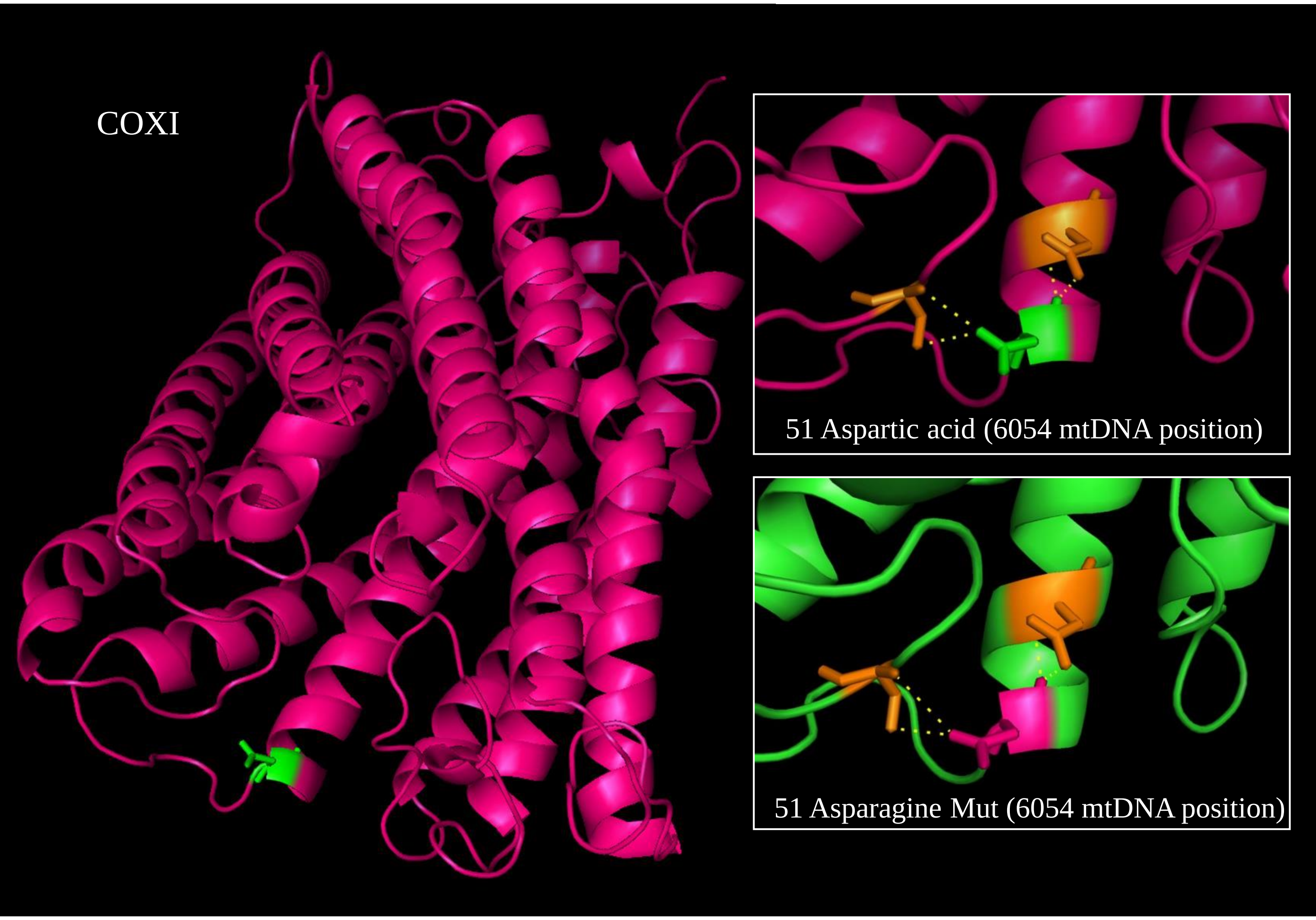
COXI
51 Aspartic acid (6054 mtDNA position)
51 Asparagine Mut (6054 mtDNA position)

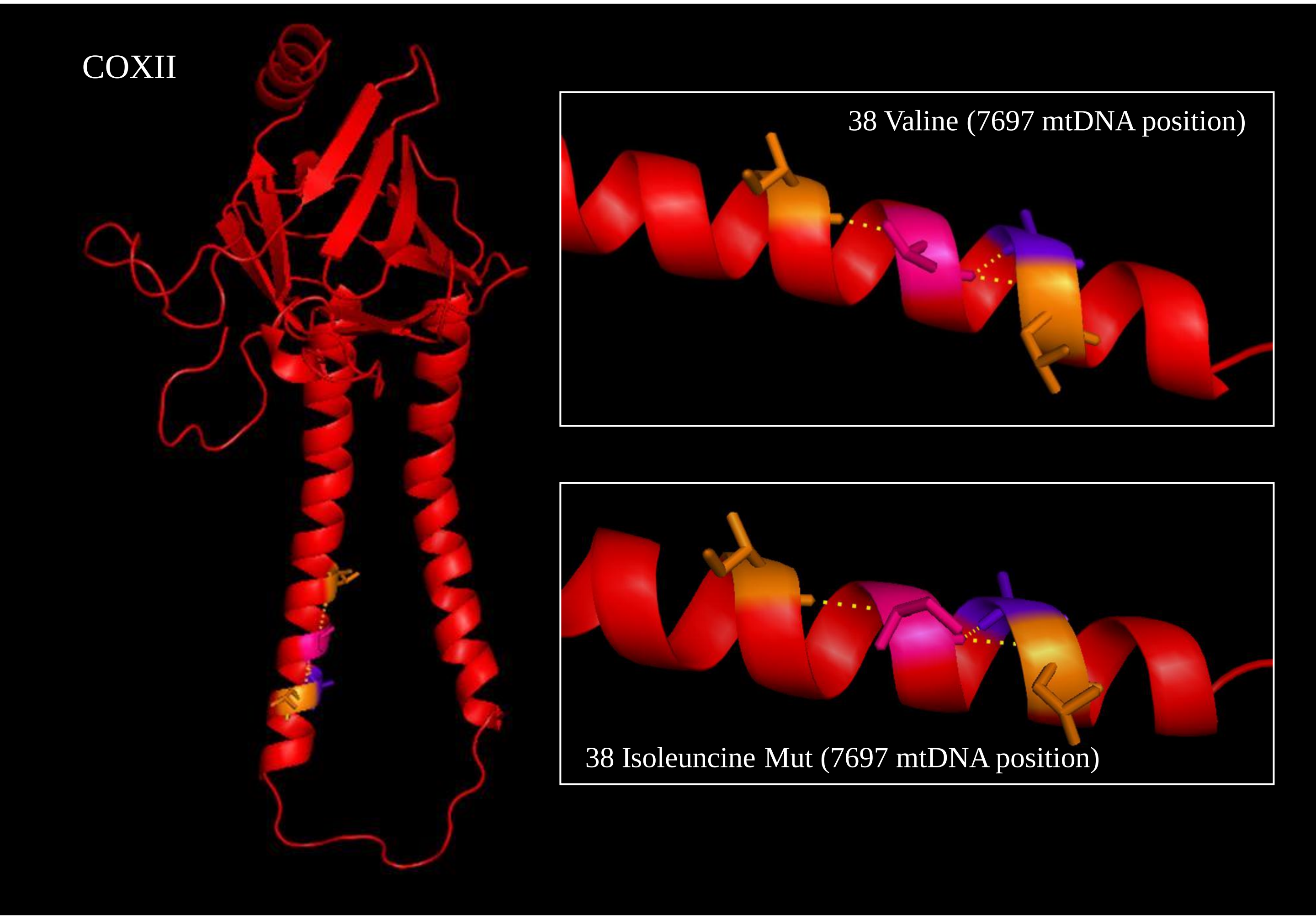
COXII
38 Valine (7697 mtDNA position)
38 Isoleuncine Mut (7697 mtDNA position)

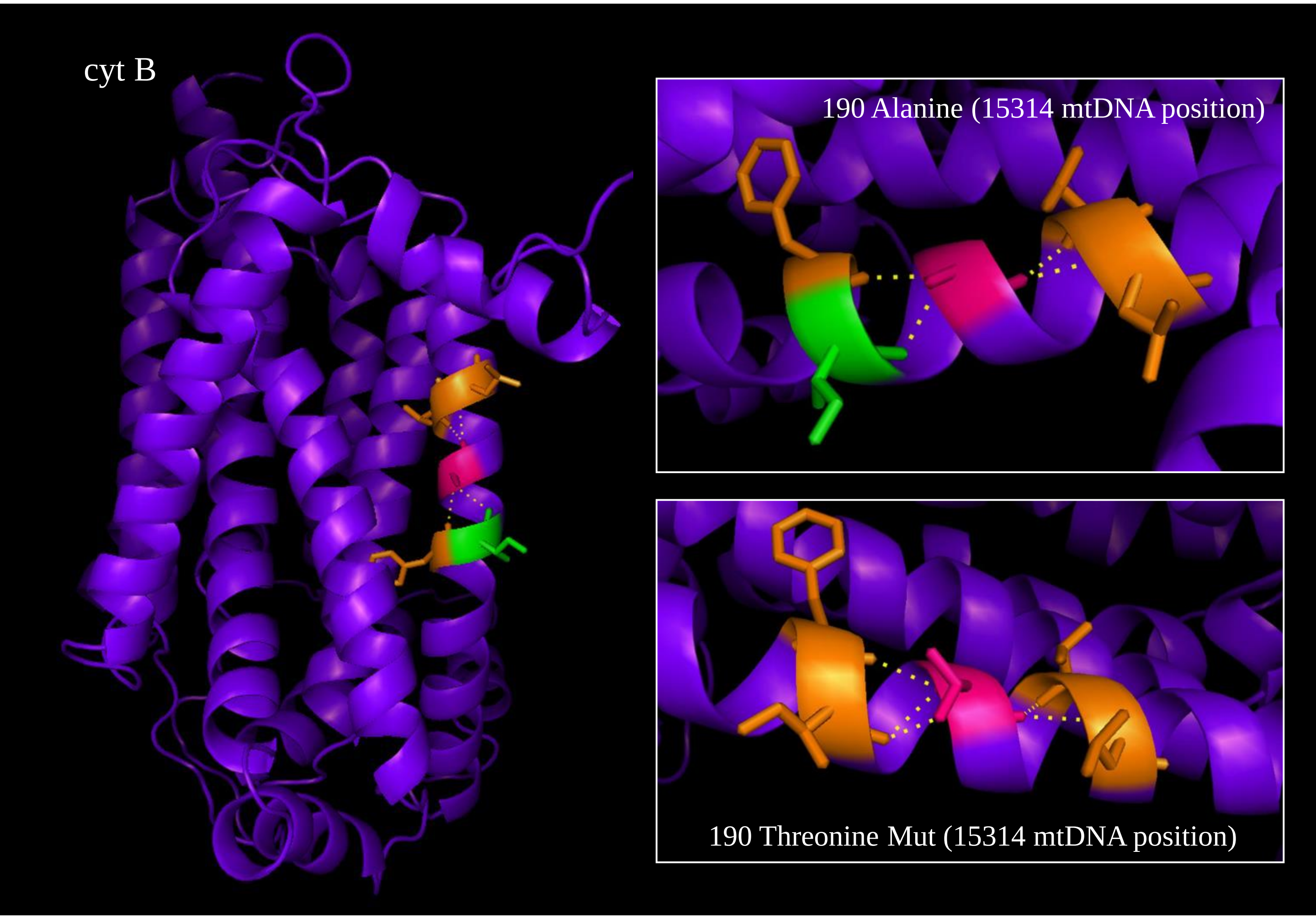
cyt B
190 Alanine (15314 mtDNA position)
190 Threonine Mut (15314 mtDNA position)
